# Bullying behaviors and intention to drop-out among nursing students: the mediation roles of sense of belonging and major satisfaction

**DOI:** 10.1186/s12912-023-01584-3

**Published:** 2023-11-08

**Authors:** Hamid Sharif-Nia, João Marôco, Pardis Rahmatpour, Kelly A. Allen, Omolhoda Kaveh, Esmaeil Hoseinzadeh

**Affiliations:** 1 Educational Development Center, Mazandaran University of Medical Sciences, Sari, Iran; 2 Department of Nursing, Amol Faculty of Nursing and Midwifery, Mazandaran University of Medical Sciences, Sari, Iran; 3 William James Centre for Research ISPA – Instituto Universitário PT & FLU Pedagogy, Nord University, Bodø, NO Norway; 4 School of Nursing, Alborz University of Medical Sciences, Karaj, Iran; 5 School of Educational Psychology and Counselling, Faculty of Education, Monash University, Clayton, Australia; 6 Centre for Wellbeing Science, Melbourne Graduate School of Education, University of Melbourne, Parkville, Australia; 7 School of Nursing and Midwifery, Mazandaran University of Medical Sciences, Sari, Iran; 8 Department of Nursing, School of Nursing and Midwifery, Gorgan Branch, Islamic Azad University, Gorgan, Iran

**Keywords:** Bullying, Nursing students, Dropout intentions, Sense of belonging, Major satisfaction, Iranian universities

## Abstract

Bullying in academic settings has long-lasting implications on students’ well-being and career prospects, particularly in fields like nursing that require a high level of interpersonal skills and emotional resilience. This study explored the relationships between experiences of bullying and intentions to drop out among Iranian nursing students, with major satisfaction and a sense of belonging serving as mediating factors. A cross-sectional research design was employed from April to June 2023. Undergraduate nursing students (n = 386) filled out a five-part questionnaire namely demographic and educational information, bullying behaviors in nursing education environments, the Belongingness scale, intention to drop out, and academic major satisfaction scale. The study confirmed that experiences of bullying positively correlated with intentions to drop out, negatively affected students’ sense of belonging and major satisfaction, and established that course satisfaction and a sense of belonging acted as mediators between bullying and intentions to drop out. The findings show the urgent need for anti-bullying interventions targeting faculty behavior and educational strategies aimed at fostering a sense of belonging and course satisfaction among nursing students.

## Introduction

Bullying is a specific form of interpersonal aggression or targeted harassment, often distinguished by an imbalance of power between a perpetrator and a victim. This category of behavior can include various subtypes such as abuse, incivility, and harassment, as well as more specialized forms like mobbing, horizontal violence, and lateral violence [1, 2]. These subtypes are not merely synonyms but represent distinct manifestations of bullying, each with its own characteristics and impact [3]. Recent research suggests that nursing students are particularly vulnerable to bullying due to the hierarchical nature of their work environment. This vulnerability is heightened for new graduates who are navigating unfamiliar settings or possess limited clinical experience [4, 5]. Despite the frequency of bullying incidents reported by nurses, it is widely believed that the actual prevalence significantly underreported [6–8]. Interestingly, some nursing students suggest they expect to encounter bullying in the profession [9, 10]. However, there is concern that these students may come to normalize bullying and inadvertently perpetuate it in the future [11].

A significant number of nursing students worldwide report experiencing bullying. For instance, over half of Turkish nursing students indicated encountering at least one form of bullying on a weekly basis over the past six months [12]. Similarly, 40% of nursing students in New Zealand reported being bullied during their clinical placements [13]. Bullying occurs in various forms and settings, involving both students and faculty in academic and clinical environments [14, 15]. The most frequently reported types of bullying during training include verbal abuse, discourteous behavior, and intimidation [12, 16]. Prior research suggests that bullying between nurses and students is more prevalent than in any other professional relationship within nursing, with teacher-to-student bullying considered the most severe [3]. Cooper et al. identified three core bullying behaviors commonly reported by nursing students: assignments or tasks given as punishment rather than for educational purposes, punitive grading, and the imposition of unmanageable workloads or unrealistic deadlines [16]. The repercussions of such bullying are severe; students undergoing clinical placements have reported experiencing anxiety, panic attacks, physical discomfort, and a significant loss of confidence and self-esteem [17]. Based on the results of a review study conducted on bullying in nursing students, classmates, professors, instructors, and hospital nurses have been reported as the sources of bullying behaviors [18].

A work environment perceived as unsafe can have far-reaching consequences, including contributing to nursing shortages, compromising patient care, and limiting educational opportunities for nursing students [3]. Empirical evidence suggests that the prevalence of bullying within the profession can lead nurses to exit the field, thereby exacerbating existing shortages and straining healthcare systems. Moreover, the psychological toll of bullying—manifested in stress, anxiety, and diminished self-esteem—can adversely affect patient care by fostering a lack of empathy and reducing the overall quality of care provided [9].

## Literature review

### Intention to drop out

Dropping out of school/university is one of the risky behaviors whose consequences affect not only the educational system but also the economy and employment of a society [19]. Dropout is recognized as a complex phenomenon that has multiple causes [20]. A systematic review by Al Zamel et al. identified bullying as a significant determinant influencing nurses’ intentions to leave the profession [21]. Similarly, a study by Martin et al. involving graduate students revealed that those who reported experiencing bullying from faculty members—through means such as belittlement, punitive actions, managerial misconduct, or exclusion—demonstrated decreased levels of engagement and a heightened inclination to withdraw from their academic programs [22]. Notably, while over half of nursing students encounter various forms of bullying from clinical instructors or their classmates during their training, it is believed that a substantial number of these incidents go unreported [23, 24]. This underreporting is often attributed to fears of receiving poor evaluations, which in turn can influence students’ intentions to leave their nursing programs [25]. In this vein, the first hypothesis endeavors to show the causal relationship between bullying experience and intention to drop-out.

### Belongingness

The concept of belonging is widely recognized as a fundamental psychological need, as articulated by scholars such as Baumeister & Leary (1995) and Maslow [26]. Within the context of nursing education, Levett et al. posit that this sense of belonging serves as a pervasive motivational force that significantly influences behavior and cognition [27]. Hagerty et al. (1992) define belonging as the subjective experience of being an integral part of various systems, including family, friends, educational institutions, workplaces, communities, and cultural groups. For nurses, a sense of belonging-particularly among peers-can act as a crucial buffer against the negative impacts of bullying. Competencies that foster a sense of belonging, such as self-efficacy and self-esteem, have been shown to contribute to resilience and coping mechanisms when bullying occurs [28–30].

Research by Li et al. has demonstrated a significant negative correlation between experiences of bullying and both academic engagement and a sense of belonging [31]. These factors were identified as serial mediators affecting the relationship between bullying victimization and academic achievement. The notion of “belongingness” is not only pivotal for academic achievement and retention but also plays a key role in the formation of social identity, well-being, positive clinical experiences, resilience, self-directed learning capacity, and self-efficacy [32].

In other hands, several studies demonstrated the belonging could reduce the students’ intention to dropout [19, 33, 34]. Magen-Nagar et al. mentioned that sense of belonging has mediator effects between teaching quality and intention to drop out among school students [19]. Among university students population, Yildirim et al. conducted the study among 3,837 international students in Germany, and found out that a high sense of belonging is correlated with higher study satisfaction and lower drop-out intention [34]. Based on the results of Soerensen study, a sense of belonging to the nursing profession was essential to support students from dropping out [20]. Since there were few studies in the field of nursing, one of this study’s hypotheses is testing the relationship between belonging and intention to drop out in nursing students.

### Academic major satisfaction

According to Rahmatpour et al., academic satisfaction is a critical factor in the educational experience [35]. Research indicates that bullying in academic settings is strongly correlated with reduced student satisfaction, increased stress, and coping difficulties [36]. Experiencing bullying has been shown to result in negative psychological outcomes, including elevated levels of depression, low self-esteem, and decreased academic satisfaction. Furthermore, the level of satisfaction with one’s academic major has been found to be significantly influenced by experiences of bullying during clinical training [37]. Nursing students are not only vulnerable to bullying but also face academic pressures that can exacerbate negative psychological responses.

In addition, according to literature, students’ satisfaction with their study and university experience could reduce their dropout rates. Duque et al. mentioned that students achieving a high level of academic satisfaction can contribute to reducing their tendency to drop out [38]. Choi et al. reported that satisfaction on study major is one of factors influencing Korean nursing students’ intention to drop out [39].

### Theoretical framework

The dropout models mentioned that the determinants of dropout include internal individual factors e.g., study satisfaction, and external factors e.g., study environment [40]. Study environment also focused on social cognitive theory. According this theory when nursing students are placed in an environment where professors or nurses perform bullying behaviors, they are likely to start modeling these behaviors [41, 42]. On the other hand, based on Maslow’s Hierarchy of Needs, when safety and security needs are met, students can move to the next level of the hierarchy, which is a sense of belonging. Therefore, if the nursing students are in an environment where the also psychological and emotional safety of the students is not established due to the bullying behavior of the people around them, they cannot experience the sense of belonging [43, 44].

### The Iranian context

According to the evidence of Pörhölä et al.‘s study, culture is an important and determining factor in the prevalence and forms of bullying among students [45]. Therefore, Iranian studies have been discussed in this section. Numerous studies have consistently highlighted job dissatisfaction and attrition among nurses because of experiencing bullying behaviors from their supervisors [46]. However, limited research has been conducted on this issue within academic settings. Notably, Iranian nursing students have reported medium to high levels of bullying in educational environments [25]. It is important to consider that the perception of cultural taboos in Iran may influence the underreporting of such incidents [47]. On the other hand, Currently, there is no proportionality between the number of nurses and hospital beds in Iran [48]. Despite the fact that a large number of nursing students are trained in the country every year, their dropout is not only financially beneficial for the system, but also affects the future nurses’ shortage.

Given the prevalence of a bullying culture in Iranian academic settings and its detrimental impact on nursing students’ educational motivation and the overall quality of education, this study aims to fill a research gap. Additionally, the study will explore the influence of physician dominance in clinical settings. To the best of our knowledge, no study has comprehensively examined these variables in tandem, as most existing research focuses either on school students or hospital nurses. Therefore, this research will investigate the interrelationships between bullying behaviors in nursing educational settings, sense of belonging, major satisfaction, and intentions to leave the profession among Iranian nursing students.

### Hypotheses

1. There is a causal relationship between bullying experience and intention to drop-out.
2. There is a negative causal relationship between bullying experience and sense of belonging.
3. There is a negative relationship between bullying experience and major satisfaction.
4. The major satisfaction and sense of belonging have mediating roles in the relationship between nursing students’ bullying experience and intention to drop-out.

## Methods

### Study design

This research employed a cross-sectional study design to explore the relationships among bullying behaviors in nursing educational settings, sense of belonging, major satisfaction, and intentions to drop out. The study focused on undergraduate nursing students from Alborz and Mazandaran Medical Sciences Universities. Data were collected through an online survey hosted on the Porsline platform (https://porsline.ir/) from April to June 2023.

A link to the survey, accompanied by a concise description of the study’s objectives, was disseminated via various social media applications, including Telegram, What’s App, and Persian applications. Participants had the flexibility to respond using smartphones, tablets, or laptops. To validate if the actual students answered the survey link, we sent questionnaire link into university official channels.

### Inclusion and exclusion criteria

Eligible participants had to meet the following criteria: (1) Be undergraduate nursing students enrolled at Alborz or Mazandaran Medical Sciences Universities who had completed at least one semester; (2) Express willingness to participate in the study; and (3) Not be concurrently employed as nursing staff.

Students who declined to participate or who failed to complete the survey in its entirety were excluded from the study.

### Participants

Based on the study’s design, which included four latent variables and 66 observed variables, a minimum sample size of 382 was required. This calculation assumed a significance level of less than 0.05, a power level of 0.8, and a small effect size of 0.2, as defined by Bakker et al. (2019) and Leppink, O’Sullivan, & Winston (2016). To account for potential dropouts or incomplete responses, a total of 400 undergraduate nursing students from Alborz and Mazandaran Medical Sciences Universities were recruited for the study. Participants were selected using a convenience sampling technique.

To validate if the actual students answered the survey link, we implemented the following strategies:

#### Informed consent

Before participating in the study, students were provided with a clear explanation of the research purpose, procedures, and potential risks and benefits. They were required to provide informed consent, indicating their understanding and willingness to participate.

- Confidentiality and Anonymity: To ensure the privacy of participants, the survey was designed to be anonymous, and no identifying information was collected. This approach encouraged students to provide honest responses without fear of repercussions.
- Distribution through Official Channels: The survey link was shared with nursing students through official channels, such as university email systems or learning management platforms. This helped to ensure that the survey reached the intended participants and minimized the risk of unauthorized access.
- Monitoring and Quality Control: We closely monitored the survey responses to identify any potential issues, such as multiple submissions from the same individual or responses that did not align with the study’s objectives. In such cases, we took appropriate measures, such as removing duplicate or irrelevant responses, to maintain the integrity of the data.

### Measurements

- **Demographic and educational information**: age, gender, semester, Grade Point Average (GPA), and source of bullying behavior (Classmate-professor-clinical instructor-nurses).
- **Bullying Behaviors in Nursing Education Environments**: A valid and reliable scale that developed by Cerit, Keskin [49]; four factors consist of “Isolation of students from the education environment” (4 items), Attack on academic achievement (4 items), Attack on personality (6 items), Direct negative behaviors (4 items); A six-point Likert scale will use to define the frequency of behaviors (0: never experienced, 1: experience for a few times a year, 2: experience for a few times a month, 3:experience for a few times a week, 4: experience once a day, and 5: experience a few times a day). The higher score means the experience more bullying behaviors.
- **The Belongingness Scale (Persian version)**: A valid and reliable scale that developed by Ashktorab, Hasanvand [32]; 3 subscales consist of “self-esteem” (13 items), “connectedness” (10 items), and “efficacy” (8 items);a 5-point Likert scale ranging from 1 (never true) to 5 (always true). Higher mean scores are indicative of higher levels of belongingness.
- **Intention to drop out**: A valid and reliable scale that developed by Ekornes [50]; eight items; a 5-point Likert scale (1 = strongly disagree; 5 = strongly agree). Higher mean scores are indicative of higher intention to drop out.
- **Academic Major Satisfaction Scale (AMSS)**: A valid and reliable scale that developed by Nauta [51]; six items, a 5-point Likert scale (1 = strongly disagree; 5 = strongly agree). The higher score means higher academic major satisfaction.

### Translation and content validity

Except for the Belongingness Scale, which was already available in Persian, all other scales were translated into Persian following the World Health Organization’s 2016 protocol for forward–backward translation techniques. Subsequently, the Content Validity Ratio (CVR) and Content Validity Index (CVI) were employed to assess the necessity and relevance of the items. These evaluations were conducted by a panel of 10 experts specializing in psychometric studies and medical education. According to Lawshe’s 1975 table, the minimum acceptable CVR value when utilizing 10 experts was 0.62. Additionally, the minimum acceptable CVI value for each individual item was set at 0.7.

### Data analysis

In a first step, to establish evidence of validity related to the internal structure of the constructs, Confirmatory Factor Analysis (CFA) was performed on the polychoric correlation matrix. The analyses utilized either the DWLS estimator for CFA or robust ML for the mediation model, both available in the lavaan package [52] for the R statistical system (The R Foundation for Statistical Computing, 2021). The study employed a range of goodness-of-fit indices to assess the model’s fit, including the Chi-square statistic (χ2), Comparative Fit Index (CFI), Tucker-Lewis Index (TLI), Root Mean Square Error of Approximation (RMSEA), and Standardized Root Mean Square Residual (SRMR). According to established criteria [53, 54], an acceptable model fit is indicated by CFI and TLI values exceeding 0.90, and RMSEA and SRMR values falling below 0.06 and 0.08, respectively.

At the second step, reliability assessment, internal consistency analyses were conducted using the “SemTools” R package [55]. Specifically, Cronbach’s alpha ordinal coefficient (α_ord) (Cronbach, 1951) and coefficient omega (ω) (McDonald, 2013) were calculated for each factor. The reliability of second-order constructs was assessed using ω_L1 (McDonald, 2013). A satisfactory level of internal consistency was indicated by alpha and omega values of 0.7 or higher, as recommended by Marôco (2021).

In the third and last step, the hypothetical mediation model was fitted and evaluated using the lavaan package, with standard goodness-of-fit indices applied for model assessment. Standard errors of model’s estimates were calculated by the lavaan’s delta method. Statistical significance tests relied on t-tests for the coefficients upon proper estimation of standard errors Additionally, R^2^ values were calculated for both mediator and criterion variables.

### Ethical consideration

Upon receiving approval for the study protocol from the ethics committee of Alborz University of Medical Sciences [Ethic code: IR.ABZUMS.REC.1401.290], the questionnaire link was shared with students. The first page of the online questionnaire included essential information such as the study’s objectives, the number of items in the survey, the estimated time required for completion, the researcher’s affiliation and contact email for inquiries, and the study’s ethical code. Participants were informed that their participation was voluntary and that their responses would be aggregated and published anonymously. The online questionnaire items were not visible to participants until they agreed to participate by clicking the “Next” button, effectively completing the online informed consent form. Informed consent was obtained from all participants.

## Results

In this study, 386 undergraduate nursing students participated. The mean and standard deviation of their age was 22.63 ± 2.2 years and their GPA was 17.26 ± 1.2 of 20. Other demographic and educational information of participants were provided in Table 1.

**Table 1:** Demographic and educational information of participants (n = 386)

variables		n	%
**Gender**	Female	252	65.2
	Male	134	34.7
**Academic year**	First year(only 2nd semester)	81	21.0
	Second year	89	23.0
	Third year	94	24.3
	Fourth year	121	31.3
**Source of bulling**	Classmate	72	18.6
	Professor	131	33.9
	Instructor	57	14.7
	Nurses	106	27.4

### Evidence of validity and reliability of constructs

Goodness of fit indices and reliability measures, as described in the methods section, for the different constructs are presented in Table 2. Overall, all constructs displayed evidence of good factorial validity and reliability assuring the proper validity and reliability of the data used in the mediation model.

**Table 2:** Goodness of fit indices and reliability measures for the latent variables used in the mediation model

Construct	Factor’s loadings range	Reliability measures	Fit indices
**Bullying** - Isolation of students from the education environment - Attack on academic achievement - Attack on personality - Direct negative behaviors	0.8 to 0.9 0.8 to 0.85 0.72 to 0.85 0.74 to 0.89 0.81 to 0.86	ω_L1_ = 0.81 α_ord_ = 0.89, ω = 0.83 α_ord_ = 0.85, ω = 0.82 α_ord_ = 0.92, ω = 0.90 α_ord_ = 0.89, ω = 0.81	CFI = 0.992 TLI = 0.991 SRMR = 0.068 RMSEA = 0.064
**Belongingness** - Self-esteem - efficacy - Connectedness	0.90 to 0.96 0.40 to 0.86 0.42 to 0.87 0.57 to 0.80	ω_L1_ = 0.92 α_ord_ = 0.88, ω=,89 α_ord_ = 0.89, ω = 0.86 α_ord_ = 0.87, ω = 0.87	CFI = 0.989 TLI = 0.989 SRMR = 0.063 RMSEA = 0.072
**Major satisfaction**	0.86 to 0.91	α_ord_ = 0.94, ω = 0.92	CFI = 0.996 TLI = 0.993 SRMR = 0.045 RMSEA = 0.127
**Intention to drop out**	0.66 to 0.90	α_ord_ = 0.92, ω = 0.89	CFI = 0.990 TLI = 0.984 SRMR = 0.074 RMSEA = 0.112

### Mediation model

**Fig. 1:**
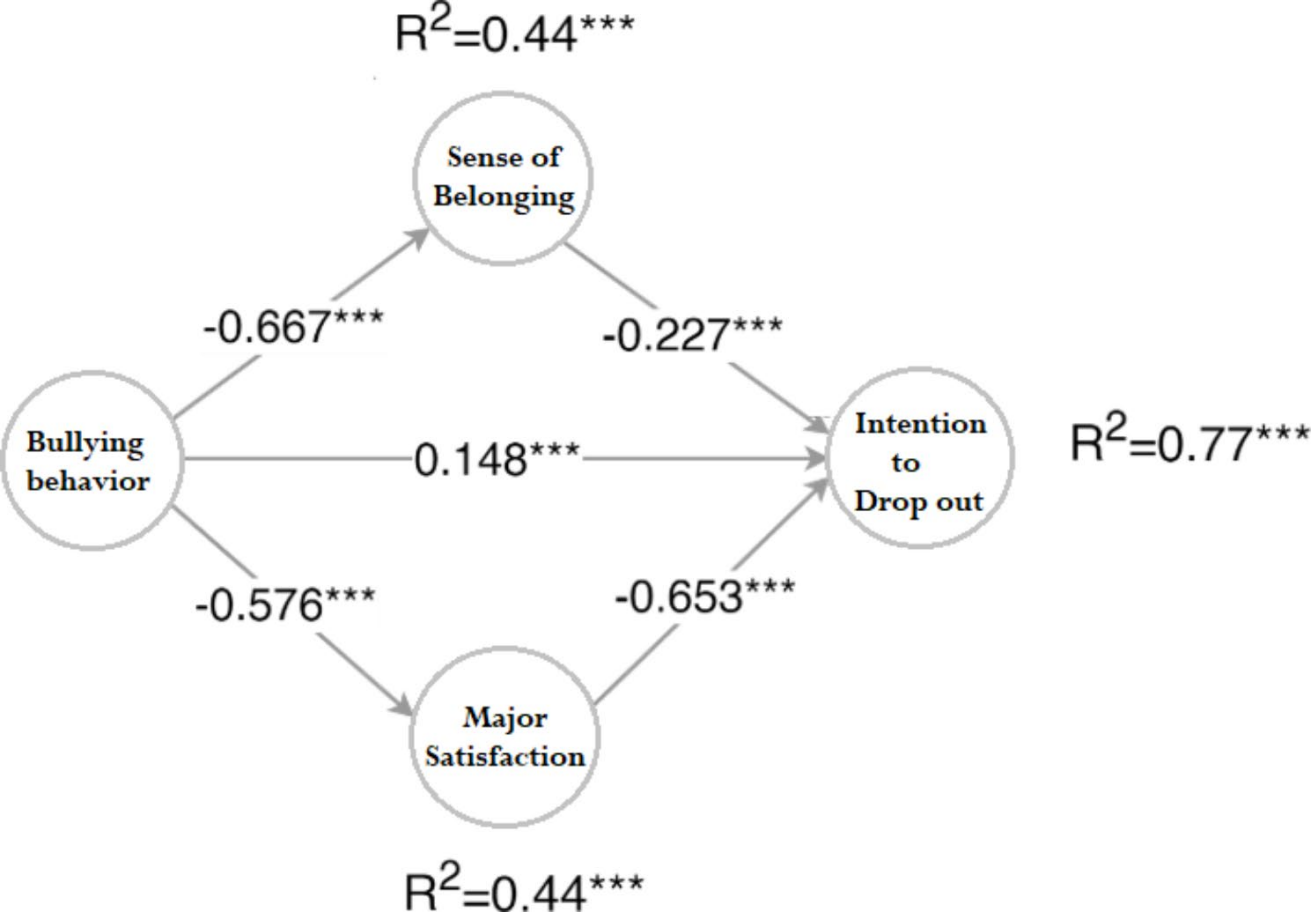
The double mediation model of bullying on the intention to drop out

The mediation model’s results are presented in Fig. 1, showing good overall fit (CFI = 0.98, TLI = 0.979, SRMR = 0.08, RMSEA = 0.07). The model explained 77.0% of the variation observed in Intention to leave (R^2^ = 0.770, p < 0.001).

Based on the results, the bullying variable directly affects the intention to drop out (β = 0.15, p < 0.001). On the other hand, the belongingness (β=-0.227; p < 0.001) and major satisfaction (β=-0.653, p < 0.001) were statistically significant related to intention to drop out. Also, bullying had a negative effect on belongingness (β=-0.667, p < 0.001) and major satisfaction (β=-0.576, p < 0.001). The bullying direct effect on the intention to drop out (β = 0.148, p < 0.001 was weaker than the indirect effects mediated trough the belongingness (β = 0.151, p < 0.001) and major satisfaction (β = 0.376, p < 0.001).

## Discussion

This study found that bullying behaviors have a direct effect on the intention to drop out among Iranian nursing students. However, the negative effects of bullying on sense of belonging and major satisfaction were stronger, and these factors indirectly affected the intention to drop out. The study highlights the importance of addressing bullying behaviors in clinical settings to improve nursing students’ sense of belonging and major satisfaction, which can ultimately reduce their intention to drop out. The following discussion points can be made based on the study results and related search results. Bullying is a significant problem in the nursing profession, and nursing students are particularly vulnerable to bullying behaviors in clinical settings [9, 14]. This can lead to decreased job satisfaction, absenteeism, tardiness, lack of teamwork, increased errors, poor communication, and collaboration [9].

The study found that bullying behaviors have a direct effect on the intention to drop out among nursing students. This highlights the need for interventions to address bullying behaviors in clinical settings to prevent nursing students from dropping out of their programs. The study also found that sense of belonging and major satisfaction mediate the relationship between bullying behaviors and intention to drop out. This suggests that interventions aimed at improving nursing students’ sense of belonging and major satisfaction may help reduce the negative effects of bullying on their intention to drop out. Related search results suggest that school connectedness and resilience may also play a role in mitigating the negative effects of bullying on mental health and life satisfaction [56, 57]. This highlights the importance of addressing bullying behaviors not only in clinical settings but also in educational settings more broadly.

Finally, related search results suggest that negative affect, core self-evaluations, and role conflict may also contribute to bullying behaviors among nurses [58]. This highlights the need for interventions aimed at addressing these underlying factors to prevent bullying behaviors in the nursing profession.

This study sought to explore the relationships between Iranian nursing students’ experiences of bullying and their intentions to leave their educational programs, with particular attention to the mediating roles of major satisfaction and sense of belonging. Notably, university professors emerged as the most frequent perpetrators of bullying. Previous research conducted in Iran corroborated these findings, indicating that over half of nursing students had encountered bullying, predominantly from nursing instructors [25, 59].

Several studies have reported that negative behavior from professors or instructors adversely impacted students’ levels of satisfaction and often led to the devaluation of nursing students. It’s worth noting that some educators may not intentionally engage in bullying behaviors, or may be unaware that their actions are perceived as such. Nevertheless, these behaviors contribute to feelings of humiliation, worthlessness, shame, and diminished self-confidence among nursing students. Moreover, punitive methods were more commonly employed than rewards in teaching practices [60, 61]. Kang found that nursing students in Australia predominantly experienced covert forms of bullying, such as exclusion and being ignored [62].

The findings of this study confirmed that experiences of bullying had a positive correlation with students’ intentions to drop out, thereby supporting Hypothesis 1 (H1). This aligns with existing literature, which indicates that bullying is a pervasive issue in academic settings and has detrimental effects on students’ attitudes toward their educational institutions, including reduced satisfaction and commitment. Exposure to bullying, whether as an observer or a victim, often leads to academic dissatisfaction and increases the likelihood of students discontinuing their programs prematurely [60]. Our results are consistent with those of Martin, Goodboy [22], who found that students subjected to bullying were more likely to consider leaving or actually exit their academic programs. Similarly, Abdollahi et al. reported that nursing students who experienced bullying in either university or clinical settings were deprived of meaningful learning opportunities, which in turn led to academic failure or withdrawal from their programs [25].

The results of the current study corroborated the negative correlation between experiences of bullying and a sense of belonging among nursing students, thereby affirming Hypothesis 2 (H2). While most existing research on the relationship between bullying and a sense of belonging has been conducted in school settings, the findings are nonetheless relevant. For instance, Li et al. demonstrated that a sense of belonging in schools served as a mediator between bullying victimization and academic achievement, effectively buffering students against the negative consequences associated with bullying [31]. Similarly, Huang found that students’ sense of belonging mediated the impact of both bullying victimization and the overall bullying climate on academic performance [63]. Studies specific to nursing students also underscore the importance of a sense of belonging in determining the quality of educational experiences [27, 32]. Patel et al. noted that bullying and incivility significantly erode nursing students’ commitment and feelings of belonging [64]. Furthermore, negative educational environments characterized by power imbalances, peer rejection, and social exclusion can undermine students’ sense of belonging [63].

The results of this study substantiated the negative correlation between experiences of bullying and major satisfaction among nursing students, thus confirming Hypothesis 3 (H3). Our findings indicate that exposure to bullying behaviors from professors, instructors, or peers significantly diminishes students’ interest and satisfaction in the nursing field. Professors and instructors serve as pivotal role models for students and are instrumental in shaping their interest in their chosen field of study. When these role models engage in bullying behaviors, it can lead to a disaffection for the nursing profession among students. This observation is supported by multiple studies, which have consistently found that bullying behaviors within educational settings adversely affect students’ perceptions of program satisfaction [36, 65, 66]. Beyond its direct impact on field-specific satisfaction, bullying also disrupts the educational process. It undermines students’ self-confidence, induces emotional distress, and contributes to overall dissatisfaction with their educational experience [36].

This study validated the mediating roles of major satisfaction and a sense of belonging in the relationship between students’ experiences of bullying and their intentions to drop out, thereby affirming Hypothesis 4 (H4). The findings elucidated the underlying mechanisms, suggesting that major satisfaction and a sense of belonging serve as key explanatory factors for why bullying in academic settings influences nursing students’ intentions to leave their programs. Marchiondo et al. posited that prolonged exposure to a bullying-prone academic environment is likely to diminish student satisfaction and elevate dropout rates [66]. Similarly, Li et al. emphasized the strong correlation between a sense of belonging and academic performance, noting that students who feel a stronger sense of belonging tend to perform better academically [31]. When students encounter a lack of respect, love, and acceptance from classmates or instructors, they are more susceptible to academic failure and are more likely to discontinue their studies. Instances of verbal or behavioral abuse, or feelings of isolation within the academic setting, can make it emotionally challenging for students to engage in their academic lives. Huang et al. underscored the critical importance of the concept of belonging in academic settings, stating that reduced feelings of belonging lead to decreased classroom engagement and, ultimately, to academic failure [63].

The implications of bullying for nursing students are manifold, extending beyond the academic setting to include clinical environments. Research has consistently highlighted the prevalence of bullying and other forms of incivility in these settings. The treatment that nursing students receive in educational and clinical environments significantly influences their attitudes toward the nursing profession and their development as nurses. If students are socialized into a nursing culture that normalizes bullying, the long-term ramifications are concerning. Such students are likely to become disengaged, anxious, or depressed professionals in the future. Moreover, these individuals may perpetuate bullying behaviors, contributing to a cycle of incivility in the workforce. This not only heightens the risk of creating unhealthy work environments but also compromises the quality of nursing care. Ultimately, these factors have a detrimental impact on patient care outcomes, as corroborated by existing research [67].

### Limitations

The limitations for the present study were: (1) The study was conducted among Iranian nursing students, which may limit the generalizability of the findings to other nursing schools or regions. (2) The study relied on self-reported data, which may be subject to social desirability bias or recall bias. (3) The study did not explore other potential factors that may affect the intention to drop out, such as academic performance, financial burden, or family responsibilities. (4) The study did not investigate the long-term effects of bullying on nursing students’ mental health, career satisfaction, or patient care outcomes. (5) The cross-sectional research design employed in this study limits the ability to draw valid causal inferences. Theoretical causal hypotheses are theory driven and basic assumption for structural equation models. Significance of estimates of the model derived from correlations between variables, support the validity of the hypothesis, but do not prove it beyond statistical confirmation.

### Implications for nurse education

The present study in nursing education found that bullying has a direct effect on the intention to drop out, but the effect is weaker than the indirect effects mediated through belongingness and major satisfaction. Belongingness and major satisfaction were also found to be statistically significant related to intention to drop out. Additionally, bullying had a negative effect on belongingness and major satisfaction. These findings suggest that addressing bullying in nursing schools and throughout a nurses’ career is crucial to prevent nurse attrition and improve clinical and financial outcomes for healthcare organizations. Strategies for addressing bullying in nursing schools and organizations may include encouraging nurses to hold each other accountable, implementing measures to prevent bullying, and empowering nurses to call out bullying behaviors.

The negative impact of bullying behavior in academic settings extends to students, educational institutions, and the broader nursing profession. Our findings underscore that nursing students subjected to bullying within university environments are at a heightened risk of discontinuing their studies. These results emphasize the critical need for anti-bullying interventions within academic settings, particularly targeting faculty behavior. Efforts to mitigate academic failure and reduce dropout rates could benefit from strategies aimed at fostering a stronger sense of belonging within the university community and enhancing major-specific satisfaction among nursing students.

## Conclusion

This study revealed positive correlations between experiences of bullying and intentions to drop out among Iranian nursing students. Furthermore, major satisfaction and a sense of belonging were identified as mediating factors in this relationship. To mitigate the impact of bullying, it is recommended that educational interventions be implemented. Firstly, nursing students should be educated about the various forms of bullying behaviors, whether they are witnesses or victims, and equipped with strategies to cope with the repercussions. University administrators should also take proactive steps to raise awareness among nursing professors and instructors about the detrimental effects of bullying on students’ academic performance. This could contribute to fostering a culture of respect and professionalism within academic settings.

## Data Availability

Due to the privacy of the research participants, the data generated during the current study are not publicly available but are available from the corresponding author upon reasonable request.

## References

[1] Tight M. *Bullying in higher education: an endemic problem?* Tert Educ Manag, 2023: p. 1–15.

[2] Kennedy RS (2020). A meta-analysis of the outcomes of bullying prevention programs on subtypes of traditional bullying victimization: Verbal, relational, and physical. Aggress Violent Beh.

[3] Seibel LM, Fehr FC (2018). They can crush you: nursing students’ experiences of bullying and the role of faculty. J Nurs Educ Pract.

[4] Courtney-Pratt H (2018). I was yelled at, intimidated and treated unfairly: nursing students’ experiences of being bullied in clinical and academic settings. J Clin Nurs.

[5] Minton C, Birks M (2019). You can’t escape it: bullying experiences of New Zealand nursing students on clinical placement. Nurse Educ Today.

[6] Al Muharraq EH, Baker OG, Alallah SM (2022). The prevalence and the relationship of workplace bullying and nurses turnover intentions: a cross sectional study. SAGE open Nursing.

[7] Johnson AH, Benham-Hutchins M (2020). The influence of bullying on nursing practice errors: a systematic review. AORN J.

[8] Qutishat M. *Underreporting bullying experiences among nursing students in clinical training sittings: a descriptive study*. Arab J Psychiatry, 2019. 30(2).

[9] Amoo SA (2021). Bullying in the clinical setting: lived experiences of nursing students in the Central Region of Ghana. PLoS ONE.

[10] Foster B, Mackie B, Barnett N (2004). Bullying in the health sector: a study of bullying of nursing students. New Z J Employ Relations.

[11] Tee S, Özçetin YSÜ, Russell-Westhead M (2016). Workplace Violence experienced by nursing students: a UK survey. Nurse Educ Today.

[12] Palaz S (2013). Turkish nursing students’ perceptions and experiences of bullying behavior in nursing education. J Nurs Educ Pract.

[13] Minton C (2018). New Zealand nursing students’ experience of bullying/harassment while on clinical placement: a cross-sectional survey. Collegian.

[14] Smith CR (2016). Seeing students squirm: nursing students’ experiences of bullying behaviors during clinical rotations. J Nurs Educ.

[15] Beckmann CA, Cannella BL, Wantland D (2013). Faculty perception of bullying in schools of nursing. J Prof Nurs.

[16] Cooper JR (2011). Students’ perceptions of bullying behaviours by nursing faculty. Issues in Educational Research.

[17] Birks M (2018). A ‘rite of passage?’: bullying experiences of nursing students in Australia. Collegian.

[18] Fernández-Gutiérrez L, Mosteiro‐Díaz MP (2021). Bullying in nursing students: a integrative literature review. Int J Ment Health Nurs.

[19] Magen-Nagar N, Shachar H (2017). Quality of teaching and dropout risk: a multi-level analysis. J Educ Students Placed Risk (JESPAR).

[20] Sørensen J, Nielsen DS, Pihl GT (2023). It’s a hard process–nursing students’ lived experiences leading to dropping out of their education; a qualitative study. Nurse Educ Today.

[21] Al Zamel LG (2020). Factors influencing nurses’ intention to leave and intention to stay: an integrative review. Home Health Care Management & Practice.

[22] Martin MM, Goodboy AK, Johnson ZD (2015). When professors bully graduate students: effects on student interest, instructional dissent, and intentions to leave graduate education. Communication Educ.

[23] Qutishat M (2019). Underreporting bullying and Harassment perceived by undergraduate nursing students: a descriptive correlation study. Int J Ment Health Psychiatry.

[24] Budden LM (2017). Australian nursing students’ experience of bullying and/or Harassment during clinical placement. Collegian.

[25] Abdollahi Z et al. *Investigation of bullying behaviors in clinical settings from the nursing students’ views* 2020.

[26] Maslow AH. *A theory of human motivation. Psychological review, 50(4), 370–396*. 10.1037/h0054346 1943

[27] Levett-Jones T (2007). Belongingness: a critique of the concept and implications for nursing education. Nurse Educ Today.

[28] Allen K, Vella-Brodrick D, Waters L. *School belonging and the role of social and emotional competencies in fostering an adolescent’s sense of connectedness to their school* Social and emotional learning in Australia and the Asia-Pacific: Perspectives, programs and approaches, 2017: p. 83–99.

[29] Allen K-A (2021). Belonging: a review of conceptual issues, an integrative framework, and directions for future research. Australian J Psychol.

[30] Arslan G, Allen K-A, Tanhan A (2021). School bullying, mental health, and wellbeing in adolescents: mediating impact of positive psychological orientations. Child Indic Res.

[31] Li L, Chen X, Li H (2020). Bullying victimization, school belonging, academic engagement and achievement in adolescents in rural China: a serial mediation model. Child Youth Serv Rev.

[32] Ashktorab T (2015). Psychometric testing of the Persian version of the belongingness scale–clinical placement experience. Nurse Educ Today.

[33] Suhlmann M, et al. Belonging mediates effects of student-university fit on well-being, motivation, and dropout intention. Social Psychology; 2018.

[34] Yildirim HH, Zimmermann J, Jonkmann K. The importance of a sense of university belonging for the psychological and academic adaptation of international students in Germany. Zeitschrift für Entwicklungspsychologie und Pädagogische Psychologie; 2021.

[35] Rahmatpour P, Sharif Nia H, Peyrovi H. Evaluation of psychometric properties of scales measuring student academic satisfaction: a systematic review. J Educ Health Promotion. 2019;8. 10.4103/jehp.jehp_466_19 PMC6967218 32002428

[36] Todd D, Byers D, Garth K (2016). A pilot study examining the effects of faculty incivility on nursing program satisfaction. BLDE Univ J Health Sci.

[37] Ren L, Kim H, Jung MS (2015). The association between bullying experience related to clinical placement and psychological well-being in nursing students. J Korean Acad Community Health Nurs.

[38] Duque LC, Duque JC, Suriñach J (2013). Learning outcomes and dropout intentions: an analytical model for Spanish universities. Educational Stud.

[39] Choi J (2021). Factors influencing nursing students’ intention to drop out. J Industrial Convergence.

[40] Mashburn AJ (2000). A psychological process of college student dropout. J Coll Student Retention: Res Theory Pract.

[41] Sidhu S, Park T (2018). Nursing curriculum and bullying: an integrative literature review. Nurse Educ Today.

[42] Swearer SM (2014). Reducing bullying: application of social cognitive theory. Theory into Practice.

[43] Levett-Jones T, Lathlean J (2009). The ascent to competence conceptual framework: an outcome of a study of belongingness. J Clin Nurs.

[44] Scheunemann A (2022). A longitudinal analysis of the reciprocal relationship between academic procrastination, study satisfaction, and dropout intentions in higher education. Eur J Psychol Educ.

[45] Pörhölä M (2020). Bullying in university between peers and by personnel: cultural variation in prevalence, forms, and gender differences in four countries. Soc Psychol Educ.

[46] Sharif Nia H (2022). The relationship between abusive supervision, psychological ownership, and quality of nursing care: the mediating role of job satisfaction. Perspect Psychiatr Care.

[47] Mamaghani EA (2018). Experiences of Iranian nursing students regarding their clinical learning environment. Asian Nurs Res.

[48] Farahani MA (2017). Attrition among Iranian nursing students: a qualitative study. Nurse Educ Pract.

[49] Cerit K, Keskin ST, Ekici D (2018). Development of instrument of bullying behaviors in nursing education based on structured equation modeling. Asian Nurs Res.

[50] Ekornes S (2022). The impact of perceived psychosocial environment and academic emotions on higher education students’ intentions to drop out. High Educ Res Dev.

[51] Nauta MM (2007). Assessing college students’ satisfaction with their academic majors. J Career Assess.

[52] Rosseel Y (2012). Lavaan: an R package for structural equation modeling. J Stat Softw.

[53] Hu Lt, Bentler PM (1999). Cutoff criteria for fit indexes in covariance structure analysis: conventional criteria versus new alternatives. Struct Equation Modeling: Multidisciplinary J.

[54] Marôco J (2014). Análise de equações estruturais: Fundamentos teóricos, software & aplicações.

[55] Jorgensen TD (2018). semTools: useful tools for structural equation modeling. R Package Version.

[56] Carney JV (2022). The role of School Connectedness in mitigating the impact of victimization on life satisfaction. Prof School Couns.

[57] Nie W, Gao L, Cui K (2022). Bullying victimization and mental health among migrant children in urban China: a moderated mediation model of school belonging and resilience. Int J Environ Res Public Health.

[58] Homayuni A (2021). Which nurses are victims of bullying: the role of negative affect, core self-evaluations, role conflict and bullying in the nursing staff. BMC Nurs.

[59] Dinmohammadi M, Peyrovi H, Mehrdad N (2014). Undergraduate student nurses’ experiences in clinical environment: Vertical Violence. Iran J Nurs.

[60] Clarke CM (2012). Bullying in undergraduate clinical nursing education. J Nurs Educ.

[61] Magnussen L, Amundson MJ (2003). Undergraduate nursing student experience. Nurs Health Sci.

[62] Kang J. Interventions for coping with bullying need further investigation and should be built into nursing curricula. Evidence-based nursing; 2018. 10.1136/eb-2018-102904 29735724

[63] Huang L (2022). Exploring the relationship between school bullying and academic performance: the mediating role of students’ sense of belonging at school. Educational Stud.

[64] Patel SE (2022). Cross-sectional study of the relationship between experiences of incivility from staff nurses and undergraduate nursing students’ sense of belonging to the nursing profession. Nurse Educ Pract.

[65] Hirschy AS, Braxton JM. *Effects of Student Classroom Incivilities on Students* New directions for teaching and learning, 2004. 99: p. 67–76.

[66] Marchiondo K, Marchiondo LA, Lasiter S (2010). Faculty incivility: effects on program satisfaction of BSN students. J Nurs Educ.

[67] Er F, Sökmen S (2018). Investigation of the working conditions of nurses in public hospitals on the basis of nurse-friendly hospital criteria. Int J Nurs Sci.

